# Dysregulated Cell Homeostasis and miRNAs in Human iPSC-Derived Cardiomyocytes from a Propionic Acidemia Patient with Cardiomyopathy

**DOI:** 10.3390/ijms24032182

**Published:** 2023-01-22

**Authors:** Mar Álvarez, Pedro Ruiz-Sala, Belén Pérez, Lourdes Ruiz Desviat, Eva Richard

**Affiliations:** 1 Centro de Biología Molecular Severo Ochoa UAM-CSIC, Universidad Autónoma de Madrid, 28049 Madrid, Spain; 2 Centro de Diagnóstico de Enfermedades Moleculares (CEDEM), 28049 Madrid, Spain; 3 Centro de Investigación Biomédica en Red de Enfermedades Raras (CIBERER), ISCIII, 28029 Madrid, Spain; 4 Instituto de Investigación Sanitaria Hospital La Paz (IdiPaz), ISCIII, 28029 Madrid, Spain

**Keywords:** propionic acidemia, PCCB, iPSC, iPSC-derived cardiomyocytes, microRNAs

## Abstract

Propionic acidemia (PA) disorder shows major involvement of the heart, among other alterations. A significant number of PA patients develop cardiac complications, and available evidence suggests that this cardiac dysfunction is driven mainly by the accumulation of toxic metabolites. To contribute to the elucidation of the mechanistic basis underlying this dysfunction, we have successfully generated cardiomyocytes through the differentiation of induced pluripotent stem cells (iPSCs) from a PCCB patient and its isogenic control. In this human cellular model, we aimed to examine microRNAs (miRNAs) profiles and analyze several cellular pathways to determine miRNAs activity patterns associated with PA cardiac phenotypes. We have identified a series of upregulated cardiac-enriched miRNAs and alterations in some of their regulated signaling pathways, including an increase in the expression of cardiac damage markers and cardiac channels, an increase in oxidative stress, a decrease in mitochondrial respiration and autophagy; and lipid accumulation. Our findings indicate that miRNA activity patterns from PA iPSC-derived cardiomyocytes are biologically informative and advance the understanding of the molecular mechanisms of this rare disease, providing a basis for identifying new therapeutic targets for intervention strategies.

## 1. Introduction

Propionic Acidemia (PA) results from the deficiency of the mitochondrial enzyme propionyl-CoA carboxylase (PCC) and is characterized by the accumulation of propionyl-CoA, metabolites of alternative propionate oxidation, and ammonia. PA is caused by mutations in the *PCCA* or *PCCB* genes that encode the two subunits of the PCC enzyme, which carboxylates propionyl-CoA to D-methylmalonyl-CoA. Propionyl-CoA is common to the pathway for the degradation of branched-chain amino acids (isoleucine, valine, threonine, and methionine), odd-chain fatty acids, and cholesterol [1].

PA is a multisystem disorder affecting every organ in the body; however, one hallmark of the disease is the complications that arise in the heart. PA may cause cardiac disease in between 22–70% of patients, depending on the cohort, manifesting as hypertrophic cardiomyopathy (HCM) [2], left ventricular non-compaction cardiomyopathy (LVNC) [3], and, more frequently, dilated cardiomyopathy (DCM) [4,5]. Furthermore, some disturbances of the cardiac electrical activity have also been evidenced with the prolongation of the QT interval of the electrocardiogram, which can appear in up to 70% of the patients, being the most typical feature [5,6,7]. In PA, QT prolongation can be accompanied by cardiac rhythm disturbances such as ventricular ectopic beats or couplets and even life-threatening arrhythmias [5,6,7]. 

It is unlikely that a single pathomechanism is responsible for driving heart disease in PA patients. There are multiple processes that have been explored, including mitochondrial dysfunction, oxidative stress, and changes in gene expression by microRNAs (miRNAs) and/or epigenetic regulation [5]. These studies have mainly been performed in a variety of experimental models, comprising in vitro models that include patients-derived fibroblasts, immortalized cell lines, or primary rodent cells, and an in vivo model [8] that also shows cardiac alterations [9,10]. In this hypomorphic PA mouse model, we have described that alterations in intracellular calcium handling could be responsible for the increased ventricular arrhythmia susceptibility of the PA animals [11]. However, new experimental tools are needed to determine why PA weakens heart function. For that purpose, we have previously generated PCCA patient-specific induced pluripotent stem cells (iPSC)-derived cardiomyocytes (iPSC-CMs) which have offered an attractive experimental platform to model PA disease [12].

In this work, we further contributed to understanding the cardiac disease pathophysiology generating PCCB iPSC-CMs from a patient with a severe cardiac phenotype, revealing profound differences compared to previously characterized PCCA iPSC-CMs, thus highlighting the importance of the development of personalized medicine for PA disease complications. 

## 2. Results

### 2.1. Generation and Characterization of iPSC-Derived Cardiomyocytes

In this study, we have used a previously described iPSC line from a PCCB patient homozygous for the mutation c.1218_1231delinsTAGAGCACAGGA; p.Gly407fs [13]. The patient presented a severe phenotype with dilated cardiomyopathy from childhood in addition to mild-moderate psychomotor retardation with central hypotonia and short stature. To validate the pathogenicity of the PCCB variant, we have also used the isogenic-control line previously generated by CRISPR/Cas9 technology [14].

**Figure 1:**
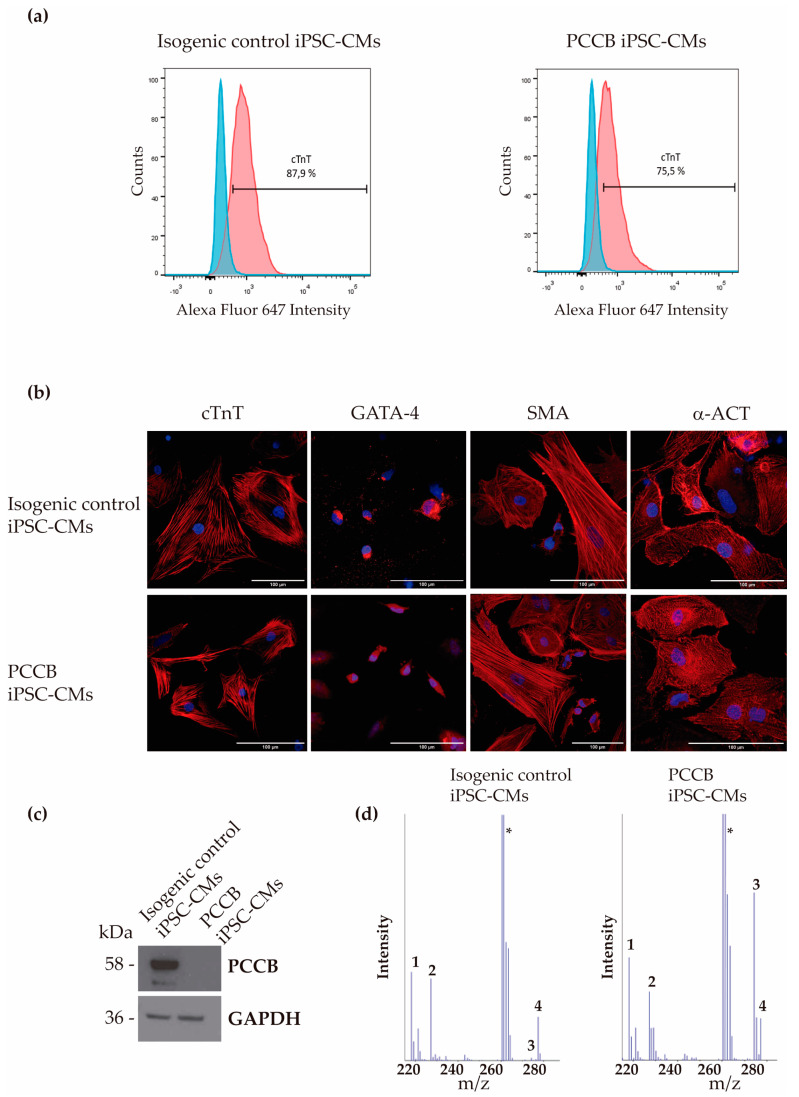
Expression of cardiac and PCCB proteins in isogenic control and PCCB iPSC-CMs. (**a**) Flow cytometry analysis for cTnT cardiac marker. A representative experiment for cTnT expression is shown. (**b**) Immunofluorescence analysis for cTnT, GATA-4, SMA, and α-ACT in iPSC-derived cardiomyocytes; scale bar: 100 µm. (**c**) Representative blot of the analysis of PCCB protein in the cardiomyocytes generated from both iPSC lines. GADPH was used as the loading control. (**d**) Acylcarnitine analysis by tandem mass spectrometry. (1) free carnitine; (2) deuterated free carnitine (internal standard); (3) propionylcarnitine; (4) deuterated propionylcarnitine (internal standard); (*) culture medium, interfering compound.

The iPSC lines were successfully differentiated into cardiomyocytes, and the efficiency of differentiation based on cardiac troponin T (cTnT) expression was analyzed by flow cytometry (Figure 1a). We found no significant differences in the efficiency for the generation of cTnT-positive cardiomyocytes in the PCCB cells (~75%) compared to the isogenic control iPSC-CMs (~85%), indicating high cardiac differentiation efficiency. In addition, immunocytochemistry showed that the PCCB and isogenic control iPSC-CMs both expressed cardiac-specific markers, cardiac troponin T (cTnT), α-smooth muscle actin (SMA), GATA4 and α-actinin 2 (α-ACT) (Figure 1b). Cardiomyocytes generated from both iPSC lines presented spontaneous beating activity, and the absence of PCCB protein (Figure 1c) and the elevated level of propionylcarnitine (2.31 µM in PCCB iPSC-CMs versus 0.06 µM in isogenic-control iPSC-CMs) was also confirmed (Figure 1d).

### 2.2. Evaluation of Cardiac Damage Markers and miRNAs

**Figure 2:**
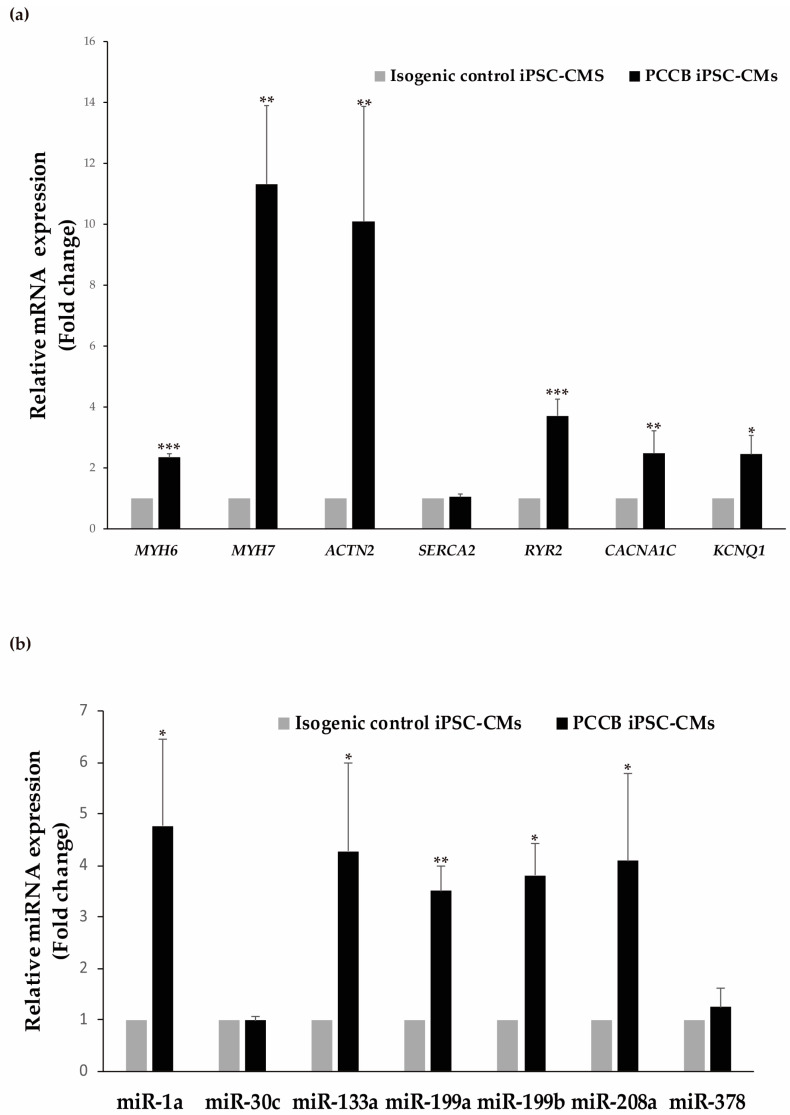
Analysis of cardiac markers and miRNAs expression in isogenic control and PCCB iPSC-CMs. (**a**) Relative mRNA expression of *MYH6*, *MYH7, ACTN2, SERCA2, RyR2, CACNA1C* and *KCNQ1* genes by qRT-PCR. (**b**) Relative expression levels of miR-1a, miR-30c, miR-133a, miR-199a, miR-199b, miR-208a, and miR-378 were evaluated by qRT-PCR in iPSC-derived cardiomyocytes. In (**a**,**b**), data represent mean ± standard deviation of three independent cardiomyocyte differentiations, at least each for triplicate. Statistical significance was determined by Student’s *t*-test. * *p* < 0.05; ** *p* < 0.01; *** *p* < 0.001.

Taking into account that the PCCB patient presented dilated cardiomyopathy, we first analyzed the expression at the mRNA level of different genes (*MYH6*, *MYH7,* and *ACTN2*) coding for cardiac damage markers (α-MHC, β-MHC, and α-actinin 2); and of several genes involved in heart calcium handling (*SERCA2, RyR2,* and *CACNA1C*) and K^+^ (*KCNQ1*) cardiac channel. Our results showed significantly increased mRNA levels of *MYH6*, *MYH7*, *ACTN2, RyR2, CACNA1C,* and *KCNQ1* genes in PCCB iPSC-CMs compared to cells from the isogenic control (Figure 2a). In addition, we selected a series of seven miRNAs expressed in cardiac tissue and reported in the literature to play a role in different processes underlying the pathogenic phenotypes of heart diseases for their analysis in our human cardiac model. Five miRNAs (miR-1a, miR-133a, miR-199a, miR-199b, and miR-208a) were significantly upregulated, showing all of them an increase in 4–5 fold in PCCB iPSC-CMs compared to the control ones (Figure 2b). It is worth noting that these miRNAs were previously found down-regulated in PCCA iPSC-CMs [12].

### 2.3. Analysis of Oxidative Stress and Mitochondrial Function 

**Figure 3:**
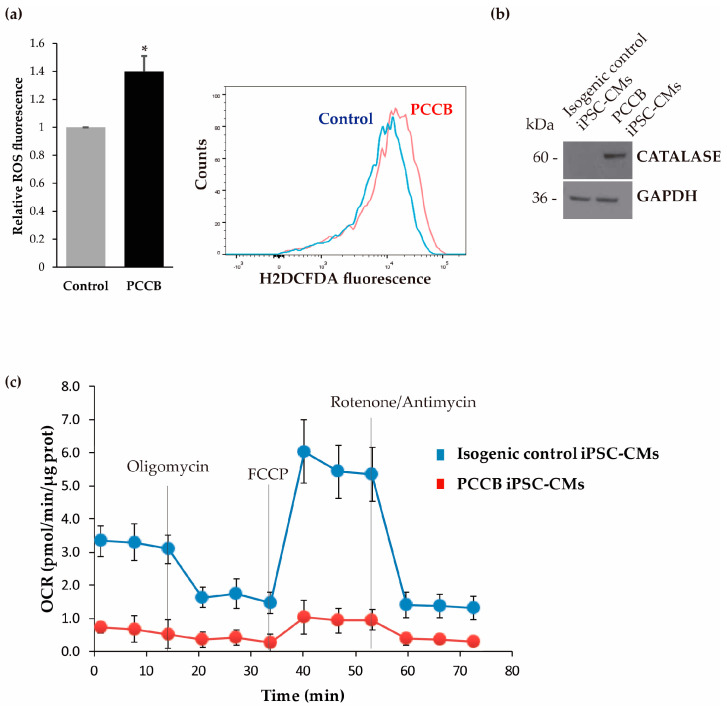
Analysis of oxidative stress and mitochondrial respiration in isogenic control and PCCB iPSC-CMs. (**a**) Detection of intracellular ROS level by flow cytometry using H_2_DCFDA as a fluorescence probe under basal conditions. Data represent mean values ± SD from at least three independent experiments. Statistical significance was determined by Student’s *t*-test. * *p* < 0.05. A representative experiment of ROS level in isogenic control iPSC-CMs (control in blue) and PCCB iPSC-CMs (PCCB in red) is shown. (**b**) Expression analysis of catalase protein by Western Blot. A representative blot is shown, and GADPH was used as a loading control. At least three experiments were performed. The corresponding quantification of proteins by laser densitometry is not shown because of the total absence of catalase enzyme in isogenic control iPSC-CMs. (**c**) Bioenergetic profile of WT and PCCB iPSC-CMs. Representative profile of basal OCR in isogenic control and PCCB iPSC-CMs after the addition of oligomycin, FCCP, rotenone, and antimycin A. The results shown are mean ± standard deviation of 3 to 5 experiments.

Our findings of the upregulation of several cardiac damage markers and miRNAs involved in differential gene expression found in pathophysiologic cardiac conditions are indicative of cardiac alterations in the PCCB iPSC-CMs. Thus, our next aim was the investigation of several potential pathomechanisms described as responsible for driving heart disease in PA. First, we examined ROS levels in control and patient iPSC-CMs by flow cytometry. ROS play an important role in cell signaling and homeostasis, and under stress conditions, ROS levels increase dramatically, resulting in damage to the cell structure and function. Increased ROS levels have been associated with SERCA2a oxidation in PA mouse hearts [11]. Our results showed that there was a ~1.5-fold increase in ROS production in PCCB iPSC-CMs (Figure 3a). We also evaluated the expression pattern of the antioxidant enzyme catalase by Western Blot analysis. As shown in Figure 3b, the expression of catalase protein was significantly increased in PCCB iPSC-CMs, which could be associated with increased ROS generation. In addition, the relative contribution of mitochondrial oxidative phosphorylation (OXPHOS) and glycolysis to ATP production in iPSC-derived cardiomyocytes was determined by measuring the overall mitochondrial respiration profile of the two iPSC-CMs lines by a flux analyzer (Figure 3c). Oxygen consumption rate (OCR) in PCCB iPSC-CMs was significantly lower than that in isogenic control iPSC-CMs indicating that energy is predominantly generated by glycolysis and that the basal mitochondrial respiration is importantly decreased. 

### 2.4. Ultrastructure Analysis and Evaluation of Autophagy Process

**Figure 4:**
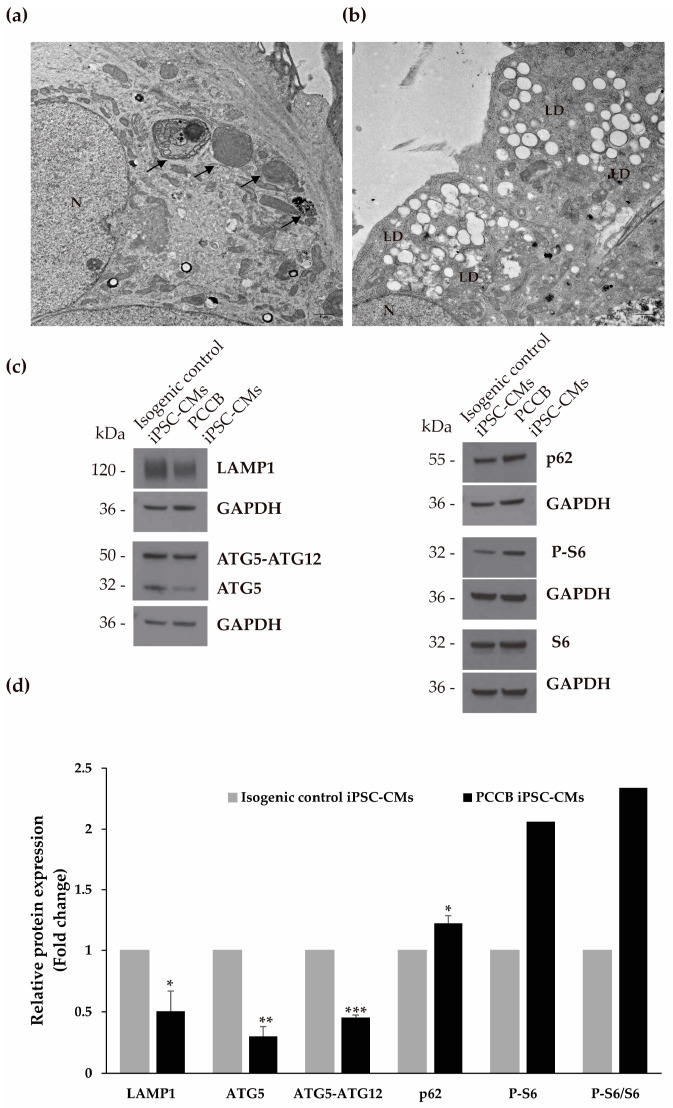
Analysis of cell ultrastructure and autophagy process in the iPSC-CMs. Representative images of EM were shown of isogenic control (**a**) and PCCB (**b**) iPSC-CMs at 3000x magnification. Black arrows show degradation vesicles (**a**). LD: lipid droplets (**b**). N: cell nucleus (**a**,**b**). Scale bar: 1 µm. (**c**) Representative blot of the protein analysis of LAMP1, ATG5-ATG12 conjugate, ATG5, p62, S6, and P-S6 (its phosphorylated form). GADPH was used as the loading control. (**d**) The corresponding quantification by laser densitometry is shown as the mean ± standard deviation of at least three experiments. In S6 and its phosphorylated form analysis, two experiments were performed (mean 0.9 and 2.06, respectively). Statistical significance was determined by Student’s *t*-test. * *p* < 0.05; ** *p* < 0.01; *** *p* < 0.001.

Our next aim was to explore native cellular landscapes by electron microscopy (EM). Ultrastructure analysis showed an increase in autophagic vacuoles in the isogenic control compared to PCCB iPSC-CMs (Figure 4a). In addition, EM images revealed a substantial accumulation of lipids droplets (LDs) in PCCB compared to isogenic control iPSC-CMs; not only many more LDs but also LDs that were much larger and distributed predominantly at the cell periphery (Figure 4b). 

In light of these observations indicating autophagy impairment, we analyzed the levels of several proteins involved in this cellular process, including LAMP1 (lysosomal associated membrane protein 1), ATG5-ATG12 conjugate which elongates the phagophore membrane to form mature autophagosome, ATG5 that initiates the formation of the autophagosome membrane and the fusion of autophagosomes and lysosomes, p62 (a marker of autophagic flux), and S6 protein regulated in the mechanistic target of rapamycin complex 1 (mTORC1) signaling pathway (Figure 4c,d). Interestingly, we observed decreased levels of LAMP1, ATG5-ATG12 conjugation, and ATG5, and an increase in p62 protein levels, along with an increase in the phosphorylated S6 protein indicating the activation of the MTORC1 pathway in PCCB compared to isogenic control iPSC-CMs. Altogether, these results suggest a decrease in autophagy in the PA patient iPSC-CMs.

### 2.5. Evaluation of Protein Expression Involved in Mitochondria-Associated Membranes, Endoplasmic Reticulum Stress, Apoptosis, and Mitochondrial Biogenesis

Next, we analyzed the expression of several proteins that reside at mitochondria-associated membranes (MAMs), including mitofusin-2 (MFN2), sigma-1 receptor (SIG-1R), and 75-kDa glucose-regulated protein (GRP75,), proteins involved in the endoplasmic reticulum (ER) unfolded protein response (UPR), including homocysteine-inducible ER stress protein (HERP) and 78-kDa glucose-regulated protein (GRP78); apoptosis regulators BCL2 and CASPASE3, along with the mRNA levels of several genes involved in mitochondrial biogenesis *PPARGC1A*, *PPARD* and *PPARG* which encode for PGC-1α, PPAR-δ, and PPAR-γ, respectively.

**Figure 5:**
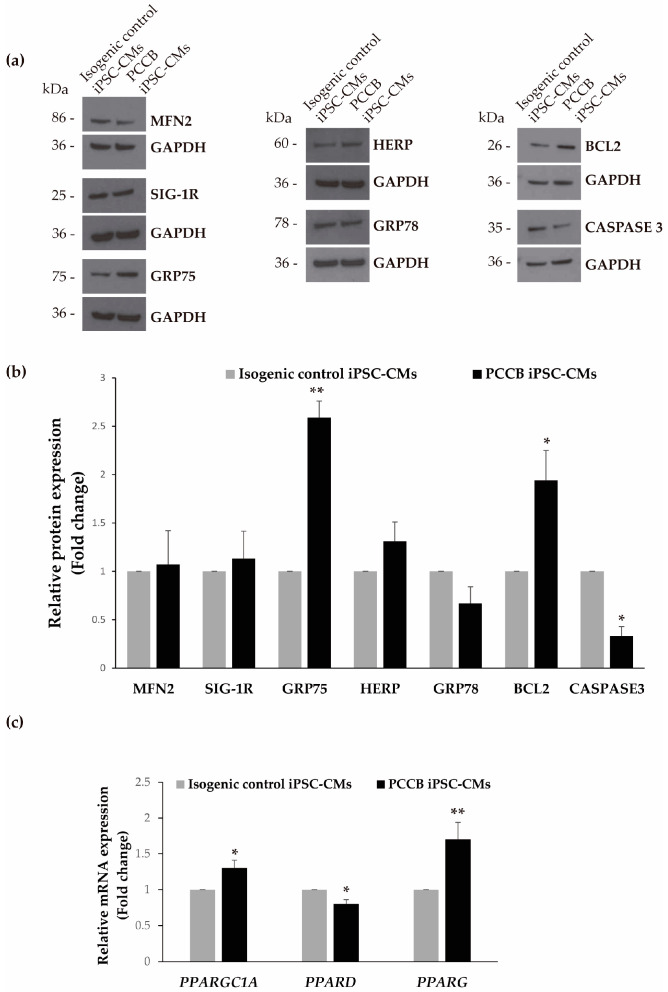
Expression analysis of proteins involved in MAMs, ER stress, apoptosis, and mitochondrial biogenesis in the iPSC-CMs. (**a**) Representative blots of the analysis of MFN2, SIG-1R, GRP75, HERP, GRP78, BCL2, and CASPASE 3 protein levels. GADPH was used as the loading control. (**b**) The corresponding quantification by laser densitometry is shown as the mean ± standard deviation of at least three experiments. Statistical significance was determined by Student’s *t*-test. (**c**) Relative mRNA expression of *PPARGC1A*, *PPARD,* and *PPARG* genes by qRT-PCR. Data represent the mean ± standard deviation of at least three experiments. Statistical significance was determined by Student’s *t*-test. * *p* < 0.05; ** *p* < 0.01.

Our results showed increased levels of GRP75 (~2.5-fold) and HERP proteins; and a decrease in GRP78 protein expression in PCCB iPSC-CMs in comparison to the control, although not reaching statistical significance (0.06) in the analysis of the latter two (Figure 5a,b). In addition, we have observed an increase in BCL2 and a decrease in CASPASE 3 protein levels in the PA patient iPSC-CMs compared to the control (Figure 5a,b). Furthermore, increased mRNA levels of *PPARGC1A* and *PPARG* and decreased mRNA levels of *PPARD* were detected in PCCB iPSC-CMs compared with its isogenic control (Figure 5c). 

## 3. Discussion

Cardiomyopathy is a well-known phenomenon in PA that may rapidly progress to death. There is an unmet clinical need to advance further in the knowledge of the precise causes that trigger cardiac complications and their progression to develop better-suited therapies for this rare disease. Although PA hypomorphic mice have provided important contributions to the understanding of PA pathophysiology, they do not fully reflect the complexity of the disease, making experimental results in this animal model very different from those applicable to humans. Considering these observations, our aim was to generate in vitro human cardiomyocytes with the same genetic background as PA patients providing a great opportunity to investigate PA cardiac complications at a human cellular level.

In this work, we have generated iPSC-derived cardiomyocytes from a PA patient with defects in the *PCCB* gene and from its isogenic control, and our results show structural defects and more pronounced signs of cardiac dysfunction than those previously observed in PCCA iPSC-CMs [12]. We have confirmed the presence of specific signs of cardiac impairment, including (i) an increase in the expression of cardiac damage markers and cardiac channels; (ii) an increase in the expression of cardiomiRNAs with relevant roles in heart diseases; (iii) increase in oxidative stress; (iv) decrease in mitochondrial respiration and autophagy; and (v) increase in the number and size of lipid droplets.

In the heart, the activity and function of cardiac-enriched miRNAs are strictly regulated to ensure proper cardiac contractility and conduction, and in pathologic conditions, dysregulation of their expression may lead to progressive heart failure [15]. Five of the studied miRNAs were found upregulated in PCCB iPSC-CMs, and their targets are involved in cellular processes altered in these cells that may aid in guiding therapeutic biological discovery. MiR-208 is involved in the late stages of cardiac development and regulates cardiac MHC expression. The observed increase in *MYH7* expression could be associated with the over-expression of miR-208a in the PA patient iPSC-CMs, leading to abnormalities in cardiac rhythm, fibrosis, and hypertrophy, as previously described [16]. In addition, increased levels of miR-208a and an increase in oxidative stress and inflammation have been observed in several types of cardiovascular patients [17]. PCCB iPSC-CMs presented miR-208 over-expression, along with increased ROS levels, which can induce the over-expression of catalase enzyme. Additional research is required to determine whether this miRNA can be considered a potential target or/and a modulator of oxidative stress in PA.

Mechanistically, miR-199a targets several genes which are crucial in autophagy and mitochondrial fatty-acid oxidation (FAO) [18,19]. These processes play a critical role in maintaining cell homeostasis. However, their impairment has been related to cardiac alterations [20,21]. Over-expression of miR-199a represses autophagy by indirectly activating the mTORC1 signaling pathway; and impairs mitochondrial FAO resulting in progressive lipid accumulation by actively repressing PPAR-δ, both lead to cardiac hypertrophy and heart failure [19,22]. In our in vitro cardiac model, we have confirmed reduced autophagy due to MTORC1 activation and to, decreased levels of lysosomal and autophagosome components; and an accumulation of lipid droplets probably caused by decreased *PPARD* levels, all of which could be related to the increased expression of miRNA-199a contributing to cardiac pathology. We can hypothesize that lipid accumulation may contribute to autophagic dysfunction in PCCB iPSC-CMs, generating a vicious circle. Moreover, the decrease in mitochondrial respiration highlights the metabolic switch from FAO to glycolysis; and mitochondrial dysfunction could be enhanced by GRP75 over-expression, which may promote MAMs formation in PCCB iPSC-CMs, as described [23]. In addition, changes in miR-199a have been related to the regulation of UPR in cardiomyocytes by controlling GRP78 expression, one of the major ER stress sensors and UPR effectors [24]. The over-expression of miR-199a may decrease GRP78 levels resulting in a protective role in PCCB iPSC-CMs. 

PGC-1α regulates cardiac energetics, and it is involved in several pathways, which include mitochondrial biogenesis, OXPHOS, and FAO. The decreased expression of PGC-1α has been postulated as an important molecular mechanism for energy starvation and metabolic defects in the failing myocardium [25]. However, the dynamics of PGC-1α expression in the failing heart may be more complex. In animal models of failure, most of the studies showed downregulation of PGC-1α [26], but some studies found no change [27]. Also, analysis of tissue samples obtained from patients at the advanced stage of heart failure showed variability of outcomes, including decreased gene or protein expression [28], unchanged gene expression [29], or even a slightly increased gene expression of PGC-1α [30]. We have observed a 1.3-fold increase in *PPARGC1A* mRNA in PCCB compared to isogenic-control iPSC-CMs, which could produce a slightly increased expression of PGC-1α protein. It is possible that this expression is not sufficient to preserve its function in the recruitment of PolII to the promoters of OXPHOS and FAO genes in PCCB iPSC-CMs, as described [27]. It would be very interesting to study PGC-1α target genes to deeply understand its target pathways in PA.

Furthermore, the elevated expression of miR-199b could lead to cardiac pathological remodeling and dysfunction in PCCB cells, as has been described in a murine model of pressure overload in which upregulation of this miRNA is sufficient to activate calcineurin/NFAT signaling [31].

Expression of miR-1 and miR-133 is coordinated and has been shown to be dramatically altered in cardiac disease, playing an important role in disease-related remodeling and arrhythmia [32,33]. The enhanced expression of these two miRNAs has been related to abnormal myocyte Ca^2+^ handling through disruption of site-specific PP2A phosphatase activity producing increased RyR2 phosphorylation and increased arrhythmogenesis phenomenon [32]. Our data have revealed the upregulation of miR-1a and miR-133a and an increased expression of mRNA *RyR2* channel, which may lead to an increased RyR2 activity resulting in the severe cardiac phenotype of the PCCB patient. For future research, it would be very interesting to analyze if the abnormally high activity of RyR2 in PA could be associated with enhanced phosphorylation of this cardiac channel. In addition, it has been shown that miR-133a has an anti-apoptotic role suppressing the expression of apoptotic proteins caspase-8, caspase-9 and caspase-3, and promoting the expression of BCL2 [34]. Our results are indicative of a regulatory effect of miR-133a on BCL2 and caspase 3 inhibiting apoptosis in damaged PCCB iPSC-CMs. Given the relevant role of miR-133 in heart disease, it is important to note the potential of this microRNA as a therapeutic target in PA.

Abnormal calcium handling and arrihythmogenic cardiomyopathy are also revealed by the presence in PCCB iPSC-CMs of increased *CACNA1C* levels, which encodes for the α-subunit of the Ca_V_1.2 LTCC. This channel is critical for the plateau phase of the cardiac action potential, cellular excitability, excitation-contraction coupling, and regulation of gene expression; perturbations of this protein have been described in a pedigree with the complex cardiac phenotype of long QT syndrome, HCM, congenital heart defects and sudden cardiac deaths [35]. It is worth noting that mRNA levels of *SERCA2* are comparable in both groups of iPSC-CMs, and no difference in protein expression was previously detected in PA hypomorphic mice; however, a higher oxidation rate in the SR-Ca^2+^ ATPase of PA mice cardiomyocytes was observed, which could be involved in the dysfunction of this protein in the PA mouse model [11]. 

Interestingly, α-actinin-2 has a dual function as both a scaffold and an interactor with signaling proteins and ion channels, which may explain why its dysfunction leads to a phenotype of simultaneous myocardial structural remodeling and ventricular arrhythmias [36]. The normal function of this protein could be impaired in PCCB iPSC-CMs due to its increased level, as has been observed in patients with distal myopathies presenting cardiac abnormalities [37].

Effects of several metabolites, including propionic acid and propionylcarnitine, on potassium currents and their channel subunits, have been analyzed in a cellular model. Remarkably, increased KCNQ1 expression was observed after chronic treatment of 10 mM propionic acid [38]. In our human cell model, increased ROS production may be associated with propionic acid-induced elevated KCNQ1 levels, as previously described [38,39].

There is an evident difference in the gene variant spectrum of PA patients from different countries and ethnic groups; however, no significant difference was found in the proportion of patients with PCCA and PCCB variants [40]. In PA, the correlations between the type and distribution of gene variants, PCC enzyme activity, and phenotype are still not very clear. Comparison of the results obtained in PCCB iPSC-CMs with those previously described in PCCA iPSC-CMs suggests different cellular alterations, probably governed by the observed dysregulation of miRNAs to be responsible, at least in part, of the different cardiac phenotype of these two PA patients. One of the clinical features of the PCCB patient was dilated cardiomyopathy, which can result in cardiac complications of heart failure and arrhythmias, both processes associated with the cellular alterations observed in our human cellular model. It is worth mentioning that signs of hypertrophy were also present in the PCCB iPSC-CMs, possibly due to the over-expression of miR-208a and miR-199a, which have been described as pro-hypertrophic miRNAs [41]. The upregulation of the studied miRNAs in the PCCB iPSC-CMs was also detected in PA hypomorphic mice and in the cardiac murine cell line HL-1 upon propionate treatment [9]. There has been growing interested in propionate biology and research efforts to understand the underlying mechanisms of severe organ dysfunction in PA. In this context, our work has revealed important findings regarding the underlying pathophysiological mechanisms in a PA-specific patient cellular model. 

Emerging miRNA therapeutics are currently offering new opportunities for health conditions, with the development of drugs to inhibit or over-express specific miRNAs that are altered in disease [42]. Due to the complex changes observed in various cellular processes regulated by multiple miRNAs, future studies will clarify whether this approach is applicable to our human cellular model of cardiomyocytes based on iPSCs differentiation. The miRNAs identified in our study, along with their predicted targets, are subject to future experimental examination that will aid in identifying novel therapeutic targets for PA.

## 4. Materials and Methods

### 4.1. Maintenance of hiPSC Lines

In this study, we have used two iPSC lines previously generated: a PA iPSC line carrying the c.1218_1231del14ins12 homozygous mutation in the *PCCB* gene [13] and the isogenic gene-corrected cell line generated through CRISPR/Cas9 gene editing [14]. Human iPSC lines were maintained on Matrigel-coated (hESC-qualified matrix, Corning, New York, NY, USA) tissue culture dishes 60 mm with mTESR^TM^ Plus medium (StemCell^TM^ Technologies, Vancouver, BC, Canadá) with regular medium changed every other day. iPSCs passaging was performed every 5 days using ReleSR^TM^ (StemCell^TM^ Technologies) at a plating ratio of 1:3. 

### 4.2. Differentiation of hiPSCs into Cardiomyocytes

HiPSCs maintained in mTESR^TM^ Plus medium was dissociated into single cells using StemPro Accutase (Gibco, Waltham, MA, USA). 1 × 10^6^ cells in 1.5 mL of mTESR^TM^ Plus medium supplemented with 10 µM Rock inhibitor (StemCell^TM^ Technologies) were seeded onto matrigel-coated 12-well plates. Cardiomyocyte differentiation was carried out using STEMdiff^TM^ Cardiomyocyte Differentiation and Maintenance Kits (StemCell^TM^ Technologies) according to manufacturer instructions. Experiments were performed after 25–30 days of iPSC-derived cardiomyocyte culture. For several analyses, cardiomyocytes were harvested using STEMdiff^TM^ Cardiomyocyte Dissociation kit (StemCell^TM^ Technologies), which includes dissociation and support medium. For methods 4.3, 4.8, and 4.9, cells were in culture for 3 or 4 additional days.

### 4.3. Immunofluorescence Staining

iPSC-derived cardiomyocytes were cultured on matrigel-coated 15 µ-Slide 8 well culture plates (Ibidi, Gräfelfing, Germany) for 5 days. Cells were fixed with Formaline Solution 10% (Sigma-Aldrich, St. Louis, MO, USA) and stained with the primary antibodies at 4 °C overnight: anti-troponin T (1:200; cTNT, Sigma Aldrich), anti-GATA-4 (1:50; GATA4, Santa Cruz Biotechnology, Dallas, TX, USA), α-smooth muscle actin (1:250; SMA, Sigma Aldrich) and anti-α-actinin (1:200; α-ACT, Sigma Aldrich). Alexa Fluor dye secondary antibodies were used (1:200). Fluorescence images were acquired using a Zeiss Confocal Fluorescence Microscope.

### 4.4. Flow Cytometry

The cultured iPSC-CMs were detached from plates by adding trypsin, and 3 × 10^5^ was used for each condition. Flow cytometry for expression analysis of Troponin T protein was performed as previously described [12]. For ROS detection, cells were cultured on matrigel-coated 12-well culture plates for 2 days, and ROS levels were monitored using H_2_DCFDA (2´,7´-dichlorodihydrofluorescein diacetate; Molecular Probes, Waltham, MA, USA) as described [43].

### 4.5. Quantitative Reverse Transcription PCR (RT-qPCR)

Total RNA was extracted from cardiomyocytes, and mRNA and miRNA analysis was performed by the procedure described in [12].

### 4.6. Western Blotting

Total protein was extracted and quantified as described [12]. The protein loading of each sample was 50 µg onto 4–12% NuPAGE™ Precast Gels. After electrophoresis, proteins were transferred to a nitrocellulose membrane in an iBlot Gel transfer device (Invitrogen, Carlsbad, CA, USA), then incubated with the primary antibodies at 4ºC overnight. The primary antibodies used in Western Blot analysis were as follow PCCB (1:1000, Santa Cruz Biotechnology, HERP (1:100, Enzo Life Science, Farmingdale, NY, USA), GRP78 (1:1000, Novus Biological, Centennial, CO, USA ), GRP75 (1:1000, Abcam, Cambridge, UK), SIG-1R (1:1000, Santa Cruz Biotechnology), MFN2 (1:1000, Abnova, Taipei, Taiwan), S6 (1:1000, Cell Signaling, Danvers, MA, USA), phosphorylated S6 (1:1000, Cell Signaling), catalase (1:1000, Abcam), BCL2 (1:500, Cell Signaling), caspase 3 (1:1000, Cell Signaling), LAMP1 (1:1000, Cell Signaling), ATG5 (1:500, Santa Cruz Biotechnology), p62 (1:2000, Novus Biologicals). The secondary antibodies were anti-mouse IgG HRP-linked (1:2000, Cell Signaling) and anti-rabbit IgG HRP-linked (1:5000, Cell Signaling). Antibody against GAPDH was used as a loading control (1:5000, Abcam). Enhanced chemiluminescence reagent (ECL, GE Healthcare, Chicago, IL, USA) was used for protein detection. Band intensity for each protein was quantified with BioRad GS-900 Densitometer (BioRad, Hercules, CA, USA) and ImageLab program (BioRad, Hercules, CA, USA).

### 4.7. Biochemical Measurements

Acylcarnitines were analyzed by tandem mass spectrometry at the Centro de Diagnóstico de Enfermedades Moleculares (CEDEM), Universidad Autónoma de Madrid, Madrid, as previously reported [44].

### 4.8. Electron Microscopy Sample Preparation and Analysis

The ultrastructure of the control and patient-derived cardiomyocytes was observed by electron microscopy, as previously described [12].

### 4.9. Mitochondrial Bioenergetics Analysis

Oxygen consumption rate (OCR) was assessed using a Seahorse XFe96 Analyzer (Agilent, Santa Clara, CA, USA). Four days before the assay, iPSC-CMs were seeded at a density of 20,000 in each well coated with Matrigel^®^ in a total volume of 80 µL of STEMdiff^TM^ Cardiomyocyte Maintenance Kit (StemCell^TM^ Technologies, ). Mitochondrial measurements were performed using the Seahorse XF Cell Mito Stress Test (Agilent 103015-100, Agilent, Santa Clara, CA, USA) according to the manufacturer’s instructions. OCR was measured following the sequential addition of 2 µM of oligomycin, 1.5 µM of carbonyl cyanide 4-(trifluoromethoxy)-phenylhydrazone (FCCP) and 0.5 µM of antimycin/rotenone. The results were normalized to the protein amount and analyzed by using the Seahorse XF96 software (Wave 2.6).

### 4.10. Statistical Analysis

All values shown are average values from *n* experiments that have been carried out independently and with different biological samples. Cardiomyocyte differentiation from iPSC lines was performed 4 times, and the analysis was carried out at least with 3 biological replicas for triplicate. The statistical significance of the differences between the analyzed groups was evaluated using a two-tailed unpaired *t*-test distribution. The differences were considered significant based on the *p*-values obtained: * <0.05, ** <0.01, and *** <0.001.

## Data Availability

The data that supported the findings of the present study are available from the corresponding author upon request.
